# Community vaccine perceptions and its role on vaccination uptake among children aged 12-23 months in the Ileje District, Tanzania: a cross section study

**DOI:** 10.11604/pamj.2016.23.162.8925

**Published:** 2016-04-06

**Authors:** Pai Elia Chambongo, Patrick Nguku, Peter Wasswa, Innocent Semali

**Affiliations:** 1 Tanzania Field Epidemiology and Laboratory Training Program, Dar es Salaam, Tanzania; 2 50 Haile Sellasie Street Asokoro,Abuja, Nigeria; 3 African Field Epidemiology Network,Kampala, Uganda; 4 Department of Epidemiology and Biostatistics, School of Public Health and Social Sciences, Muhimbili University of Health and Allied Sciences,Dar es Salaam, Tanzania

**Keywords:** Vaccination uptake, perception, Ileje

## Abstract

**Introduction:**

Underutilization of vaccines still remains a challenge in many regions across the world. Ileje district is one of the districts in Tanzania with consistently low pentavalent vaccine uptake (69%) and with drop out of 15%. We determined the vaccination completion with regard to Oral Polio virus, Measles, Bacillus Calmette-Guérin, and pentavalent vaccines and its association with community perceptions on vaccines.

**Methods:**

We conducted a cross sectional study in Ileje district from October to December 2013. We sampled 380 mothers using a multistage random sampling technique. We analysed data using EPI INFO. We summarized descriptive variables using mean and standard deviation and categorical variables using proportions. We conducted bivariate and multivariate logistic regression to identify factors influencing vaccination uptake, statistical significance was assessed at 95% confidence interval.

**Results:**

Mean age of the mothers was 27 years (SD 6.5 years) while that of their children was 16 months (SD 3.6 months). Fully vaccinated children were 71.1% and partially vaccinated were 28.9%, 99.2% were vaccinated with BCG vaccine and 73.4% were vaccinated with all OPV vaccine. Predictors of vaccination completion included negative perception on the vaccine provider-client relationship (AOR 1.86, 95%CI1.03-3.35), Perceived satisfaction with vaccination services (AOR 2.63, 95%CI 1.1 - 6.3). Others include child being born in the health facility (AOR 13.8 95% CI 8.04-25.8) and younger age of a child (AOR 0.51, 95%CI 0.29-0.9).

**Conclusion:**

Improving quality of vaccination services, promoting health education and sensitizing community on health facility delivery will improve child vaccination completion in the district

## Introduction

Childhood vaccination is a highly effective method for preventing vaccine preventable diseases[1–4]. Globally it is estimated that about 22.4 million children under one year of age were not vaccinated with a third dose of pentavalent vaccine (Diphtheria-Pertussis-Tetanus-Hepatitis B-Haemophilus type b influenza vaccine (DPT-HB-Hib)) in the year 2012, of whom 70% live in Africa [5] while 0.54%of the unvaccinated children were found in Tanzania. Children who miss vaccines or are partially vaccinated are said to be at high risk of vaccine preventable diseases that claim millions of lives each year [6, 7]. It is also estimated that 10 million children under-five years die, one third of which is attributable to infectious diseases that would have been vaccine prevented [8, 9]. A child is said to underutilize a vaccine if he or she misses out on one or more of the recommended vaccine dose [10]. In response to the underutilization of vaccines, World Health Organization (WHO) and United Nation for International Children and Education Funds (UNICEF) developed the Global Immunization Vision and Strategy (GIVS) in 2006 aimed to help countries to vaccinate more people, especially those who are hard to reach [11]. Furthermore, GIVS emphasized on reaching every district strategy (RED), as it showed improvement in raising vaccination coverage in some countries where it was practiced [12]. RED focuses on ensuring that each district achieves 90% coverage for all antigens by 2015, thus all 156 districts in Tanzania should have 90% of its eligible children vaccinated. Unfortunately, only 50% of the districts in 2012 reported to have vaccination coverage above the required target (90%) [13]. Ileje district is among the districts with vaccination uptake consistently below 90% over the past ten years with its most recent third dose pentavalent coverage being 69% with a dropout of 15% [13]. Vaccination uptake is influenced by various factors that include social and economic status of the family [14, 15], mother health status and experience in utilization of health care services [16, 17], age and education of the mother [18–20], polygamies [21], physical factors like distance to the facility [22], area of residence [23, 24] and facility factors including lack of vaccines and diluents [25], staff shortage [26], lack of policy on immunization [27], lack of adequate transport facilities [12] and type of care providers [28]. Parents are sometimes reluctant to send their children for vaccination [29]. Lack of access to vaccination services could also be due to mothers’ perception of vaccines. Perceiving of vaccine as safe and protective is likely to have positive effect on demand for vaccination [30, 31]. In 2003, the summit of independent European vaccination experts was held with the aim of addressing the perception of vaccines and limited vaccination utilization to the general public [32]. Community perception that vaccines were deliberately contaminated with anti-fertility agents and Human Immune deficiency Virus (HIV) were observed to be major barrier towards polio eradication in Nigeria [33, 34],as a result, fewer children were sent to vaccine centres [35]. Other factors related to underutilization of childhood vaccines are concerns on the vaccine safety and potential vaccine adverse effects [36–38]. It has also been observed that the greater the perception that a vaccine is associated with adverse effects, the lower the vaccine demand in the community [30, 39]. It is also reported that health providers’ poor attitudes also contribute significantly to the negative perception on the immunization services among communities leading to low utilization and low vaccination among children [40, 41]. There are also observations that the severity of vaccine preventable diseases (VPD) influence positively or negatively on vaccination uptake [39, 42, 43]. In spite of the above likely explanatory factors, there is limited data on community perception of vaccines and vaccination and its relationship with vaccine uptake in countries like Tanzania. Thus the study assessed the role of community perception on vaccination uptake among children in Ileje District, Tanzania.

## Methods

**Study area and population:** we conducted a cross-sectional study in Ileje District in Mbeya Region. The district has a total population of 148490, of which 4.2% are infants, 18.9% are children under five and 22.7% are women of child bearing age ( National Census 2012). Also the district has a total of 26 health facilities including 23 dispensaries, 19 of which are owned by the government and 4 are owned by faith-based organisations. There is only one government-owned health centre, two hospitals, one of which is government-owned while the other is owned by a faith-based organization. The district is divided into two divisions, which are further divided into nine wards. A ward sub-divided to four or five villages.

**Sample and procedure:** we conducted a cross sectional study among mothers with children aged 12 to 23 months in Ileje district. A sample size of 380 mothers with children aged 12 -23 months was calculated by using a formula for calculating sample size for cross-sectional study, assumed a design effect 1.5, precision 5% and 69% proportion of children vaccinated with third dose of pentavalent as reported from the district [13]. Multistage sampling was done, started with selection of two divisions. In the second stage, we randomly selected two wards from each division. At the third stage, we randomly selected two villages in each ward. This was followed by the listing of all children 12-23 months residing in the selected villages. From this list, we randomly sampled children in each ward proportional of the total village population of all the selected villages.

**Data Collection:** a structured questionnaire was initially developed in English, which was later back-translated in Kiswahili language was used in the interview. It was pre-tested so as to identify difficult terminologies, ambiguous questions, estimated time for filling one questionnaire and other questionnaire related problems. This interviewer-administered questionnaire was used to collect data on participants’ demographic information, children vaccination information and mother perceptions on vaccines and vaccination services. Child vaccination information was obtained from mother's history that was verified using the child vaccination card.

**Study Variables:** the outcome was whether a child had completed the under-five vaccination schedule. A child was considered to have completed vaccination schedule if one had received one dose of Bacillus Calmette-Guérin (BCG), one dose of measles vaccine, four doses of Oral Polio Vaccine (OPV) and three doses of Pentavalent (DTP-HB-Hib). It was coded ‘1’ if completed and ‘0’ otherwise. The explanatory variables that explained variation in immunization status included demographic factors and community perceptions on vaccination. Statements on vaccine perception was read to respondents who were asked to respond to each statement by either strongly agreeing, agreeing, disagreeing and strongly disagreeing with the perception statements. The perception statements covered the following categories on vaccination: perception toward vaccines efficacy, vaccine safety, provider-client relationship and severity of the vaccine preventable diseases. Perception responses were then reduced to binary variables whereby those responded strongly agree and agree were categorized as positive and coded ‘0’ the rest categorized as negative and coded ‘1’. Agreeing or disagreeing with the perception statement grouped mothers into whether they perceived vaccination positively or negatively respectively.

**Data Management and Analysis:** we collected data from October to November 2013 using a structured closed-ended questionnaire. The collected data was checked for completeness, consistency and other errors at the end of each data collection day. Data was then entered into an Epi-info database. It was followed with data cleaning and analysis was done using EPI INFO software version 3.5.4 Descriptive analysis was performed to determine frequency distribution of demographic factors, antigen uptake and perceptions. Bivariate analysis was performed to determine the crude odds ratio (cOR) of the associations between independent and dependent variables, where statistical significance was set at 0.05. All variables that had a p<0.2 at binary analysis were included into final multivariate logistic regression model which determined the association between vaccination uptake with independent variables.

**Ethical Considerations:** the proposal and request for ethical and research clearance was submitted to the Muhimbili University of Health and Allied Science (MUHAS) Institutional Review Board (IRB) which granted clearance to conduct the study. Before a participant was recruited into a study she was asked to give a written signed informed consent. Confidentiality of the information as explained in the informed consent form was highly maintained. No non authorized individual was allowed to access information without permission, no name or postal address of any participant was collected.

## Results

**Baseline characteristic of the respondents:** A total of 380 mothers with children aged between 12 months to 23 months were recruited into the study. The mean age of the mothers was 27 years (SD 6.5) while that of children was 16 months (S.D 3.6). Majority of mothers 327/380 (86.1%) were married, 325/380 (85.5%) of mothers had completed seven or less years of schooling, 95/380 (25.1%) of the mothers had given birth before 21 years and 10/380 (2.6%) had given birth at the age of 41 years or older (Table 1).

**Table 1:** Characteristics of study participants in Ileje district, Tanzania, Dec 2013 (N=380)

Variable	Number of respondents	Percentage
**Mother age group at birth (years)**		
<21	95	25.1
21-30	200	52.6
31-40	75	19.7
41+	10	2.6
**Marital status**		
Married	327	86.1
Not Married	53	13.9
**Mother completed education (years)**		
≤ 7	325	85.5
>7	55	14.5
**Father completed education (years)**		
≤ 7	322	84.7
>7	58	15.3
**Mother occupation**		
Not employed	366	96.3
Employed	14	3.7
**Mother religion**		
Christian	373	98.2
Others	7	1.8
**Sex of a child**		
Female	197	51.8
Male	183	48.2
**Cld place of delivery**		
Health facility	238	62.6
Home	142	37.4

**Vaccination uptake:** High proportion of children 71.1% (95%CI 66.2% - 75%) had completed their vaccination schedule. Utilization of individual vaccines varied widely; it was highest for Bacillus Calmette-Guérin (BCG) vaccine 99.2% (95%CI 97.5% - 99.8%), followed by DPT-HB-Hib 91.8% (95%CI 88.6% - 94.4%) and least for OPV 73.4% (95%CI 68.7% - 77.8%). About eight children (2.1%) had never been vaccinated with OPV and pentavalent vaccines while 6.1% had never been vaccinated with measles vaccine. Out of 380 mothers, 376 (98.9%) had their child's vaccination card (Table 2).

**Table 2:** Levels of vaccination Uptake in children, Ileje district, Tanzania, Dec 2013 (N=380)

Variable	Number of respondents	Percentage (%)	95% CI
**Having vaccination card**			
Yes	376	98.9	
No	4	1.1	
**BCG**			
Yes	377	99.2	97.5% - 99.8%
No	3	0.8	0.2% - 2.5%
**OPV vaccinated doses**			
4	279	73.4	68.7% - 77.8%
3	56	14.7	11.4% - 18.8%
2	29	7.6	5.3% - 10.9%
1	8	2.1	1.0% - 4.3%
None	8	2.1	1.0% - 4.3%
**DPT-HB-Hib vaccinated doses**			
3	349	91.8	88.6% - 94.4%
2	17	4.5	2.7% - 7.2%
1	6	1.6	0.6% - 3.6%
None	8	2,1	1.0% - 4.3%
**Measles**			
1	357	93.9	90.3% - 95.6%
None	23	6.1	4.0% - 9.1%
**Vaccine completion**			
Complete	270	71.1	66.2% - 75.5%
Incomplete	110	28.9	24.5% - 33.8%

**Mothers perceptions on vaccines and vaccination:** About 232/380 (61.1%) mothers reported negative perceptions on the relationship between vaccine provider and clients while a greater proportion 336/380 (88.4%) reported being satisfied with the quality of vaccination services. Similarly, a high proportion 358/380 (94.2%) reported positive perception on the safety of the vaccines. However, a lower proportion 299/380 (78.6%) reported positive perception on the efficacy of the vaccines (Table 3).

**Table 3:** Mother's perceptions on vaccine and vaccination in Ileje district, Tanzania, Dec 2013 (N=380)

Perception Variables	Number of respondents	Percentage (%)
**Perceived quality of vaccine provider client relationship**		
Negative	232	61.1
Positive	148	38.9
**Perceived satisfaction with vaccination services**		
Satisfied	336	88.4
Unsatisfied	44	11.6
**Perceived safety of vaccines**		
Negative	22	5.8
Positive	358	94.2
**Perceived efficacy of vaccines**		
Negative	81	21.4
Positive	299	78.6
**Perceived severity of the vaccine preventable disease**		
Negative	120	31.6
Positive	260	67.4

**Analysis of factors associated with vaccination completion:** The final model showed that mothers who negatively perceived vaccine provider-client relationship (AOR 1.86, 95%CI 1.03-3.5) and those who were satisfied with vaccination services (AOR 2.63, 95%CI 1.1-6.3) were more likely to have their children completely vaccinated. A child being born in the health facility strongly increased the odds of being completely vaccinated (AOR 14.4, 95% CI 8.04-25.8), while being young (≤ 17 months) reduced the likelihood of being completely vaccinated (AOR 0.51, 95%CI 0.29-0.9) (Table 4).

**Table 4:** Analysis of factors associated with vaccination completion, Ileje district, Tanzania, Dec 2013 (N= 380)

Demographic variable	Vaccination status		cOR ( 95%CI)	
	Complete	Incomplete		AOR (95% CI)
	N (%)	N (%)		
**Mother age (years)**				
< 30	201(70.5)	84(29.5)	0.9 (0.53-1.51)	
30+	69(72.6)	26(27.4)	1	
**Sex of a child**				
Male	127(69.4)	56(30.6)	0.86 (0.55-1.33)	
Female	143(72.6)	54(27.4)	1	
**Child age (Months)**				
<17	133(65.8)	69(34.2)	0.58 (0.37-0.91)	0.51 ( 0.29-0.9)
17+	137(77)	41(23)	1	1
**Mother marital status**				
Married	229(70)	98(30)	0.68 (0.34-1.36)	
Unmarried	41(77.3)	12(22.7)	1	
**Mother education level**				
≤7	228(70.2)	97(29.8)	0.73 (0.37-1.42)	
>7	42(76.4)	13(23.6)	1	
**Father education**				
>7	49(84.5)	9(15.5)	2.4 (1.1-5)*	
≤7	221(68.6)	101(31.4)	1	
**Mother occupation**				
Employed	14(87.5)	2(12.5)	2.95 (0.66-13.2)	
Not employed	256(70.3)	108(29.7)	1	
**Place of birth**				
Health facility	215(90.3)	23(9.7)	14.7 (8.52-25.4)	14.4 (8.04-25.8)
Home	55(38.7)	87(61.3)	1	1
**Perceived quality of vaccine provider clients relationship**				
Negative	175(75.8)	57(24.2)	1.7 (1.1-2.7)	1.86 (1.03-3.5)
Positive	95(64.2)	53(35.9)	1	1
**Satisfaction with vaccine services**				
Satisfied	245(72.6)	91(27.4)	2.11 (1.1-4.05)	2.63 (1.1-6.3)
Unsatisfied	25(55.8)	19(44.2)	1	1
**Perception of vaccine safety**				
Negative	15(68.2)	7(31.8)	0.88 (0.35-2.23)	
Positive	255(71.2)	103(28.8)	1	
**Perceived efficacy of the vaccines**				
Negative	54(66.6)	27(33.4)	0.75 (0.44-1.27)	
Positive	216(72.2)	83(27.8)	1	
**Perceived severity of the vaccine preventable diseases**				
Negative	79(65.5)	41(34.5)	0.7 (0.44-1.11)	
Positive	191(73.5)	69(26.5)	1	

## Discussion

This study aimed to determine vaccine completion and its association with community's perception on vaccine among children aged 12-23 months in Ileje district, Tanzania. The study revealed a moderately high vaccination completion (71.1%) but with variations in vaccine uptake level among antigens. Being satisfied with vaccination services and having negative perceptions about vaccine provider-client relationship was associated with increased likelihood of completing vaccine. Other factors significantly associated with completion of vaccination were a child being born in the health facility and age of the child. Mother's perceptions on vaccine efficacy and safety and severity of vaccine preventable disease did not seem to influence child vaccination uptake Perceived satisfaction with vaccination services was seen to influence children vaccination completion. Satisfaction on vaccination services could have been as a result of good quality of vaccination service which also might have been contributed by facility factors like staff availability, short service waiting time, strictness in adherence to procedures [19]. Such attributes might have improved the quality of vaccination service and hence inspired mothers to take their children for vaccination. Similar findings were reported by the study conducted in rural in Chad [44] and in rural Nepal [45] that child vaccination uptake also depended on the quality of vaccination services. Improving quality of vaccination services might increase the likelihood of service utilization and hence higher chances of vaccination completion. Although the majority of mothers were satisfied with vaccination services, most had a negative perception on the relationship with the vaccine providers. In spite of this, they were more likely to complete vaccination than those who positively perceived vaccination. Vaccines providers can play an important role in ensuring that mother bring their children for vaccination. The way vaccine providers behave might promote or discourage mothers from sending children for vaccination. Our findings are not supported by studies elsewhere like Africa and Asia including Malawi and Ethiopia, India and Philippines [46] which revealed that mothers who had negative perception on the health providers were less likely to complete vaccination than those who perceived positively.

In the case of this study, perceiving health providers negatively could make mothers adhere to instructions closely to avoid uncomfortable encounters with providers. Thus fear and desire to maintain harmony could have made mothers send their children to health facilities and consequently finishing vaccination schedule. Respondents’ socio-demographic factors such as age of the child and whether child was born in the health facility were significantly associated with children vaccine completion. It found that older children had significantly higher likelihood of vaccination completion compared to younger children. Similar results were obtained in studies conducted in Temeke district-Tanzania (unpublished) and in Guangzhou China, these studies indicated that younger children were less likely to have been vaccinated with measles antigen and influenza vaccine compared to older children [47]. Possible explanations could be children who do not complete vaccination at the expected age had to attend at older ages to complete vaccination, the older the child the more opportunities for vaccination hence the higher the odds of vaccination completion. Another explanation could be younger children represent a cohort that experienced a period of vaccine unavailability at health facilities and consequently reduced likelihood of completing their vaccination schedule [25]. Similarly children who were born in the health facility were more likely to have completed vaccination compared to those who were delivered at home. Children born in health facilities completed vaccination schedule simply because they had more chances of getting all essential health services at birth including vaccination doses and thus were more likely to be vaccinated with BCG and OPV0 vaccines that would have been missed out by those children born at home. Increasing the number of health facility deliveries might promote positive vaccination uptake and hence result in high vaccination completion. In addition, the contexts that enabled facility delivery also enabled access to health facility for vaccinations later in life. Similar results were reported in the study conducted in rural in Nigeria by Regina Eziuka [48], rural Mozambique [49] and in Mosul Iraq [28] that delivering in health facilities increased the likelihood of vaccination completion than home deliveries. The study had some limitations, it collected information based on the child vaccination cards and history for those mothers who did not have the cards, and were required to remember number of vaccines doses their child received. There were some perception statements that required mothers to recall the time they visited vaccination clinics and yet some of the mothers might have been not able to remember the vaccination status of their children and/or the way they attended vaccination clinics. However, the majority had vaccination cards and it was believed that mothers’ responses were accurate and provided satisfactory information. The study employed a cross-section study design; therefore it was not able to infer the temporal sequence between vaccination uptake and mother's perceptions.

## Conclusion

Vaccination uptake in children aged 12-23 in Ileje showed variations per antigen; there was high utilization in BCG vaccine and low utilization in OPV vaccine. The vaccination completion was explained by mother's perception on vaccine provider-client relationship, satisfaction with vaccination services, place of delivery and age of a child. Immediate interventions to maintain and avert factors lowering vaccine uptakes level are required. Therefore the Council Health Management Team (CHMT) should ensure that the quality of vaccination services is maintained. Vaccinators should behave in that promotes the quality of vaccination service provision. The CHMT should encourage the community to deliver in health facilities and discourage child home delivery.

### What is known about this topic

- Vaccination uptake with regard to family social and economic factors;
- Vaccination uptake with regards to physical and administrative factors.

### What this study adds

- Child vaccination uptake with regards to mother's perceptions on vaccine and vaccination.
